# Characterising malaria connectivity using malaria indicator survey data

**DOI:** 10.1186/s12936-019-3078-2

**Published:** 2019-12-23

**Authors:** Carlos A. Guerra, Daniel T. Citron, Guillermo A. García, David L. Smith

**Affiliations:** 1 Medical Care Development International, 8401 Colesville Road, Suite 425, Silver Spring, MD 20910 USA; 2 Institute for Health Metrics and Evaluation, University of Washington, 2301 Fifth Avenue, Seattle, 98121 USA

**Keywords:** Malaria connectivity, Malaria importation, Malaria indicator survey, Human mobility, Human movement, Human travel

## Abstract

Malaria connectivity describes the flow of parasites among transmission sources and sinks within a given landscape. Because of the spatial and temporal scales at which parasites are transported by their hosts, malaria sub-populations are largely defined by mosquito movement and malaria connectivity among them is largely driven by human movement. Characterising malaria connectivity thus requires characterising human travel between areas with differing levels of exposure to malaria. Whilst understanding malaria connectivity is fundamental for optimising interventions, particularly in areas seeking or sustaining elimination, there is a dearth of human movement data required to achieve this goal. Malaria indicator surveys (MIS) are a generally under utilised but potentially rich source of travel data that provide a unique opportunity to study simple associations between malaria infection and human travel in large population samples. This paper shares the experience working with MIS data from Bioko Island that revealed programmatically useful information regarding malaria importation through human travel. Simple additions to MIS questionnaires greatly augmented the level of detail of the travel data, which can be used to characterise human travel patterns and malaria connectivity to assist targeting interventions. It is argued that MIS potentially represent very important and timely sources of travel data that need to be further exploited.

## Background

A crucial and legitimate concern of malaria control programmes is the threat of malaria importation [1]. It has long been acknowledged that the past failures to eradicate malaria were in part explained by an underestimation of the role of human movement on the spread of malaria infections [2, 3]. Parasites are transported within vectors as they move about in the environment to satisfy their biological needs [4, 5] and within human hosts as they travel or migrate [6, 7]. Malaria incidence and mosquito data from areas with heterogeneous transmission show that risk around mosquito habitats declines by more than 90% after approximately 1 km, suggesting the spatial scales for characterising heterogeneity in local entomological risk are around 500–700 m [8]. Human movement, on the other hand, commonly exceeds distances of 10 km or more, and represents the single most important driver of parasite dispersal in elimination settings because it transcends the limits of mosquito flight ranges [3, 7, 9]. Even considering the long-distance spread of mosquitoes through accidental anthropogenic transportation [10] and wind-borne migration [11], parasite movement by humans far outweighs parasite movement by mosqutioes across large geographical distances. Human mobility is a growing phenomenon driven by the expansion and sophistication of transport networks to satisfy an ever increasing demand of short-term travellers [10], compounded by longer-term movements as populations respond to environmental, political and economic pressures [2, 12].

As malaria retreats, transmission is progressively confined to discrete foci, and sources and sinks for parasite transmission become more apparent [13]. Malaria transmission sources can be defined as areas where the local reproductive number is above one ($R_c >1$) and hence are able to sustain endemic malaria without importing any infections. Malaria transmission sinks are areas where $R_c<1$ and are unable to sustain transmission unless parasites are imported from sources [6]. Parasite flows on a landscape, referred to as malaria connectivity, and identification of sources and sinks requires having well-resolved pictures of both malaria and human mobility patterns. Human movement data sets that can account for these connections, however, are surprisingly scarce and usually are not routinely collected as part of surveillance systems [14, 15].

Malaria indicator surveys (MIS) were developed to measure key indicators of progress on malaria control and elimination [16]. By definition, MIS are individual-level surveys conducted at the household that provide information on the general population rather than on those seeking health care. Their frequency varies greatly according to the setting from sporadic, such as when they are used to provide baseline information, to periodic, as when they are used for monitoring and evaluating malaria control [16]. MIS can capture a wide range of information that can be tailored according to the specific needs of the control programme perusing these data. They can contribute significantly to the knowledge base of malaria epidemiology at country, regional and global scales and represent a key source of medical intelligence of both programmatic and scientific relevance. Over the past two decades, many MIS have been conducted on representative population samples in a large number of malaria endemic countries to gather information about malaria prevention and treatment practices, health seeking behaviour, knowledge about malaria and malaria morbidity and mortality. Many MIS use biomarkers to test individuals for anaemia and for the presence of *Plasmodium* parasites in their blood and MIS data are increasingly used to provide input data to geostatistical models of malaria prevalence and other metrics [17–20]. Some MIS also incorporate a travel component whereby individuals are asked about their history of travel within a specified period of time [21–28].

This paper discusses the utility of MIS in the study of malaria connectivity. It argues that MIS are a convenient tool for routine collection of human travel data. The experience on Bioko Island, Equatorial Guinea (EG), is used as an illustrative example of a real world application of MIS travel data for this purpose. The terms human movement, human mobility and human travel are used interchangeably to refer to the *periodic circulation* of people, defined as humans moving away from their place of residence for more than 24 h with eventual return to it [2]. This type of movement is discussed because it is particularly relevant in the context of malaria elimination on Bioko Island, without neglecting the importance of other types of human mobility at different temporal and spatial scales  [2, 9, 29].

## The need to characterise malaria connectivity

Two critically important questions for malaria control programmes are: (i) where are the transmission sources and sinks?; and (ii) how are they interconnected? [6]. If programmes knew the *local* force of infection (FOI), defined as the rate of infection for a person who spent all their time in one place, they would have a strong basis for targeting vector control to reduce malaria. Since people move around, it is difficult to know where anyone actually acquired malaria, even with good entomological data on exposure. High quality, highly resolved data describing mosquito populations are expensive and rarely done. An alternative is to combine information about *P. falciparum* parasite rate (*Pf*PR) and human travel. Going from measures of local *Pf*PR to local FOI requires knowing something about the relationship between incidence and prevalence, which is confounded by human travel and malaria case management [6, 13, 30]. An accurate picture of local transmission thus requires having an accurate model of malaria connectivity, which in turn requires building quantitative models to estimate three-way relationships between the local FOI, local prevalence and human travel patterns.

To become infected, a human host must be present in an area when mosquitoes are actively biting. Therefore, the question for malaria transmission is not just time spent, but time spent weighted by mosquito biting activity, called time at risk, *p* [6]. More specifically, there is a need to estimate what fraction of a person’s time at risk *here* (indexed by *i*) is spent *there* (indexed by *j*). To fully quantify the time at risk, $p_{i,j}$, in the context of human travel, knowing the frequency of travel to a specific destination is as important as knowing the time spent at that location. In order to describe how this translates into risk of malaria infection, a measure of local FOI, $h_i$, at each location is also needed, which quantifies the intensity of transmission. Accounting for both travel patterns and time at risk while travelling is achieved by calculating an effective FOI as an average of the rates at which a person becomes infected at each travel destination, $h_j$, weighted by the fraction of time spent at each location, $p_{i,j}$:

$$\begin{aligned} h'_i = \sum _j p_{i,j} h_j. \end{aligned}$$

The quantity $h'_i$, the effective FOI, determines the malaria incidence that one observes for a member of the population who lives at *i*. It is only true that $h'_i =h_i$ in people who spend all of their time at location *i*.

Another useful way of describing malaria transmission is the malaria connectivity matrix, whose elements describe the number of cases over some time period *t* acquired there by people who live *here*. Using a time at risk matrix, *P*, whose elements are $p_{i,j}$, and a vector describing the local FOI, $\vec h$, whose elements are $h_i$, the effective FOI is given by the equation:

$$\begin{aligned} \vec h' = P \vec h. \end{aligned}$$

Instantaneous connectivity can also be defined as a set of per-capita infection rates, which is the matrix product of *P* with the diagonal matrix describing the FOI, $\text{ diag }(\vec h)$

$$\begin{aligned} P \cdot \text{ diag }(\vec h). \end{aligned}$$

While the elements of this matrix describe the instantaneous rates that determine connectivity, it is also useful to have a description of the number of cases as the FOI varies over time $\vec h(t)$. Let $H_i$ denote the number of residents of each geographical sub-region, and $\vec H$ the vector for the region over some time interval (from 0 to *τ*):

$$\begin{aligned} \Xi (\tau ) = \int _0^\tau \text{ diag }(\vec H) \cdot P \cdot \text{ diag }(\vec h(t)) \; dt. \end{aligned}$$

Over a year, the on-diagonal elements of $\Xi$ describe the annual number of cases acquired at home. The rows describe cases acquired elsewhere by people who live here. The row sums thus describe malaria *importation*, and the column sums describe malaria *exportation*. In a fully defined model of malaria spatial dynamics, this construct provides a useful way of both estimating where to target interventions and estimating the number of cases prevented by this targeting.

## The example of Bioko Island

Bioko, the main island of insular EG, is located around 40 km off the coast of Cameroon, comprises an area of about 2000 km^2^ and accommodates the country capital, Malabo. Malaria transmission on Bioko Island is perennial, with a long rainy season that runs between April and September. The entomological inoculation rate was extremely high by the time malaria control interventions were established in 2004, with estimates of around 900 infected bites per person per year [31]. In that year, vector control and other interventions were scaled up across Bioko, resulting in significantly reduced malaria prevalence on the island over the subsequent 15 years [30, 32]. Thanks to this, Bioko has seen a substantial reduction in malaria parasite prevalence in children, from 43.3% in 2004 to 10.5% in 2016, though this decline has stalled in the last years [32]. To monitor progress, MIS are conducted annually on a representative sample of the whole island population, with $\approx {5000}$ households visited and $\approx {14,000}$ individuals surveyed every year. Crucially, the MIS questionnaire includes a section on travel history that specifically investigates recent travel (i.e. travel that happened in the 8-week period preceding the survey) to destinations on and off the island. In addition, all consenting individuals are tested for malaria parasites using rapid diagnostic tests, making it possible to estimate *Pf*PR. Together, these circumstances present an opportunity to evaluate the utility of MIS data for investigating malaria connectivity on Bioko Island.

### Using MIS data to study human mobility and malaria

**Fig. 1:**
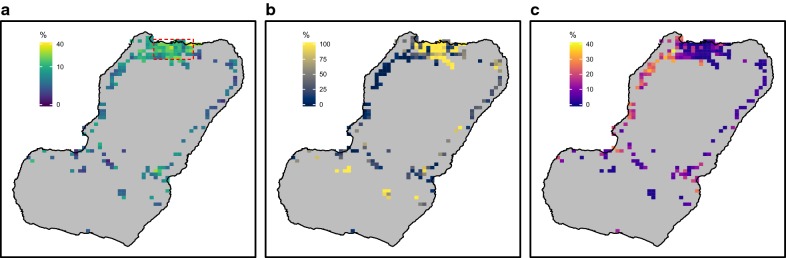
Human travel and malaria on Bioko Island. **a** Estimated percentage of people travelling to mainland EG within 8 weeks preceding the MIS. **b** Travel fraction, or the fraction of *Pf*PR that could be explained by infection importation from mainland EG. **c** Local residual transmission, or the estimated *Pf*PR in the absence infection importation. The maps were reproduced from data published in Guerra et al. [30] and are based on the 2015–2017 MIS data from Bioko Island. Pixels correspond to $1 \times 1$ km inhabited areas. The red, dashed rectangle in **a** delimits urban Malabo

Three separate studies have quantified the relationship between prevalence of malaria and human travel on Bioko Island using MIS data and all have found that the odds of malaria infection in travellers to mainland EG are between three to four times that of individuals with no such travel history [30, 32, 33]. The most recent study explored spatial relationships between malaria prevalence and human travel and found substantial, fine-grained patterns (Fig. 1) [30]. The odds of having travelled to mainland EG given any malaria infection were highly variable across the island (Fig. 1a). Through in-depth analyses and modelling it was possible to infer the fraction of the observed *Pf*PR that could be explained by infection importation from mainland EG, called the *travel fraction*. A very strong spatial pattern was noticed with high fractions evident mostly in and around Malabo, where most of the off-island travel originates (Fig. 1b). Estimates of *local residual transmission* were marked by a similarly strong spatial pattern with higher estimated transmission along the West coast, where travel to the mainland is also less frequent (Fig. 1c). In all, the study suggested high spatial heterogeneity in malaria transmission and strong malaria connectivity between Bioko and mainland EG.

**Fig. 2:**
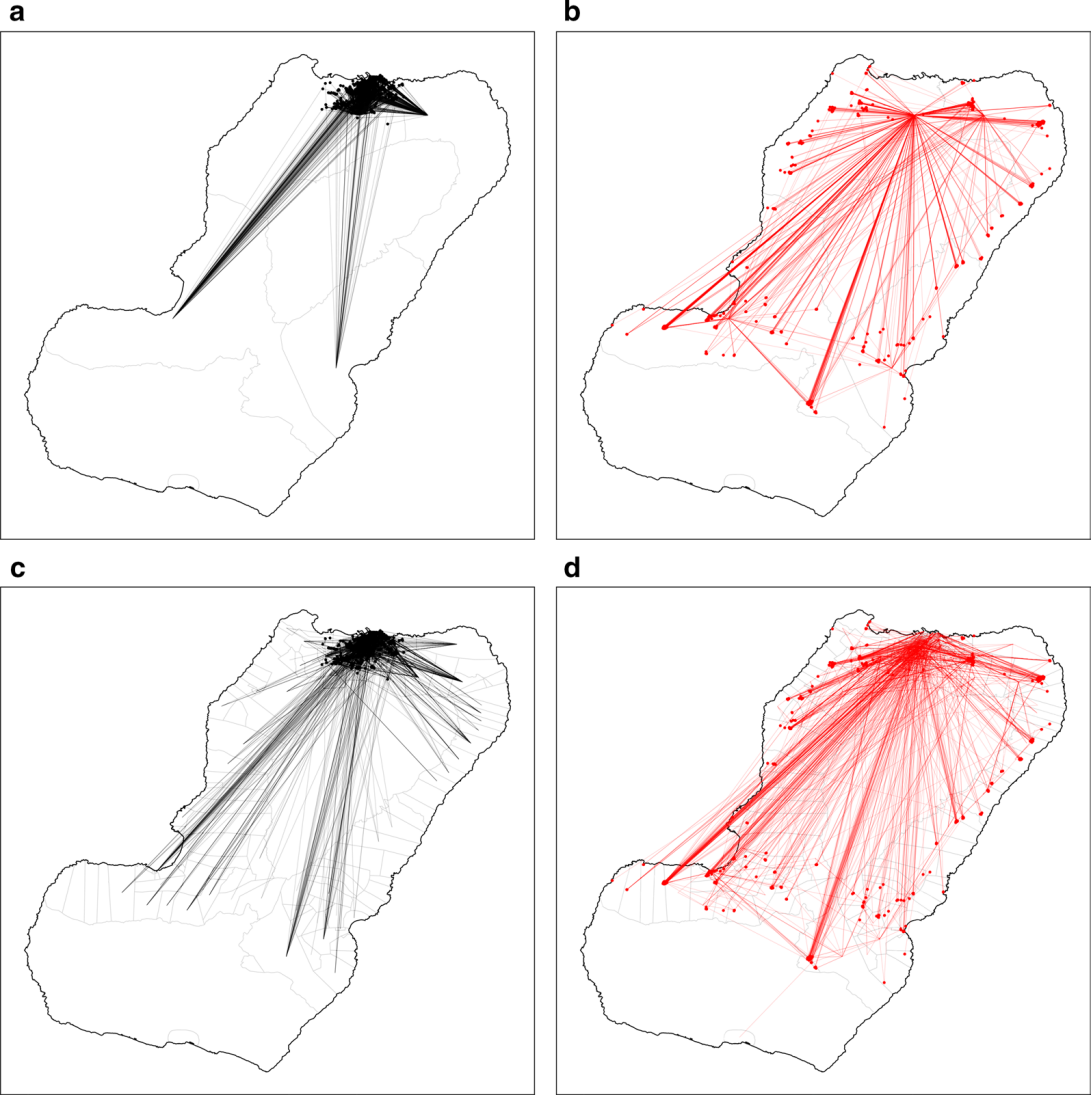
On-island travel by Malabo and non-Malabo residents. The lines connect the household of residence (dots) to the district centroid (second administrative level; **a**, **b**) and to the community centroid (fourth administrative level; **c**, **d**). The left panels illustrate travel by Malabo residents and the right panels travel by non-Malabo residents. The maps illustrate the substantial gain in detail regarding travel destinations, from four districts (top panels) to 151 communities (bottom panels)

One of the findings of this study was that the MIS data had important limitations for rendering an accurate description of these connections. First, there was limited spatial detail regarding travel destinations, which were recorded broadly, meaning that while it was possible to know where travellers were bound from at a very high spatial resolution, it was not possible to know where they were bound to with a similar level of detail. For example, when looking at travellers leaving Bioko, the answers regarding their destinations were limited to only four options: travellers bound to mainland EG, to other islands of EG, to other countries in Africa and to other destinations outside of Africa. Though the vast majority of off-island trips (84%) were made to mainland EG, there was no specific information on the final destination within the continental region. Similarly, when looking at on-island travel, the possible destinations were recorded only at the second administrative level, to one of the four island districts (Fig. 2). Second, the travel data had limited level of detail regarding frequency of travel and lacked information on the duration of trips. The answers to how many trips individuals had made were binned into a small number of categories (1–3 trips, 4–9 trips and >10 trips), rendering the information of limited use, and no question asked how many nights travellers spent away from home during trips. Third, the analyses identified the need for information on the professional occupation of travellers as well as on the means of transportation used to travel to mainland EG (i.e. air or boat travel) in order to identify high risk groups of travellers and better characterise their transport links with the mainland [34, 35].

The questionnaire of the 2018 MIS was modified to address these limitations. First, question loops were generated to characterise travel events separately and, for each, individuals were prompted to provide the community of destination on Bioko (i.e. fourth administrative division) and the district of destination in mainland EG (i.e. second administrative division). Using administrative divisions rather than locations for this purpose was considered pragmatic to simplify responses and minimise recall bias while avoiding the complexity of geo-referencing locations [36]. Second, the disaggregation of travel events allowed the calculation of frequency of travel (i.e. by recording the number of trips within the specified period) and the duration of trips was captured by asking individuals the number of nights spent away from home during each travel event. Third, the occupation of individuals was recorded and classified according to job sector and, for off-island travellers, a question on means of transportation was added.

**Fig. 3:**
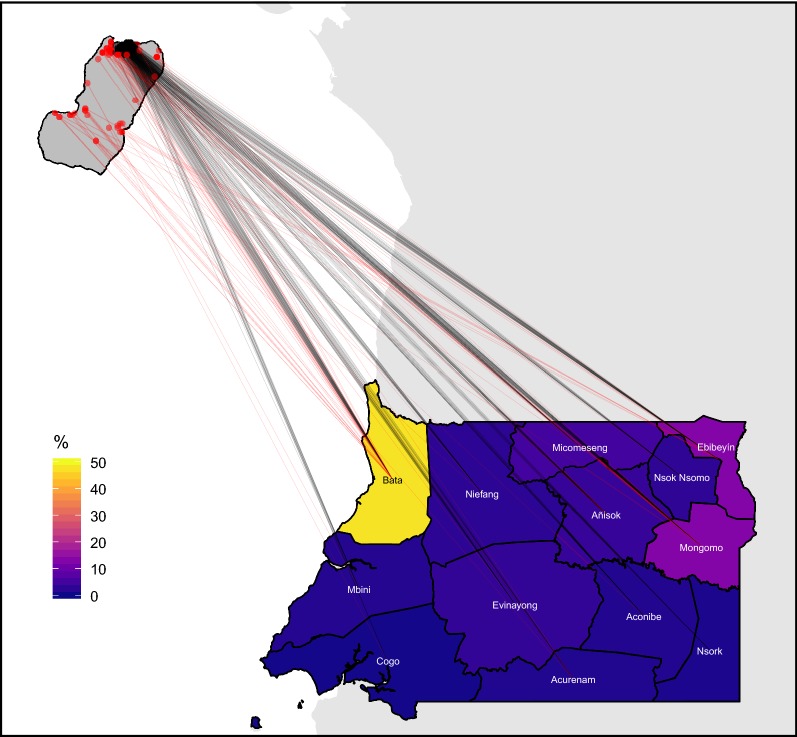
Frequency of travel by Bioko residents to districts in mainland Equatorial Guinea. The colour scale illustrates the percentage of travellers bound to each district. The lines connect the area of residence (dots) to each destination district centroid. Black and red vectors and dots illustrate travel by Malabo and by non-Malabo residents, respectively. The great majority of trips to mainland (1378/1577, 87.4%) were made by Malabo residents. Previous MIS data treated the whole continental region as a single destination

### Improved MIS travel data for characterising malaria connectivity

**Fig. 4:**
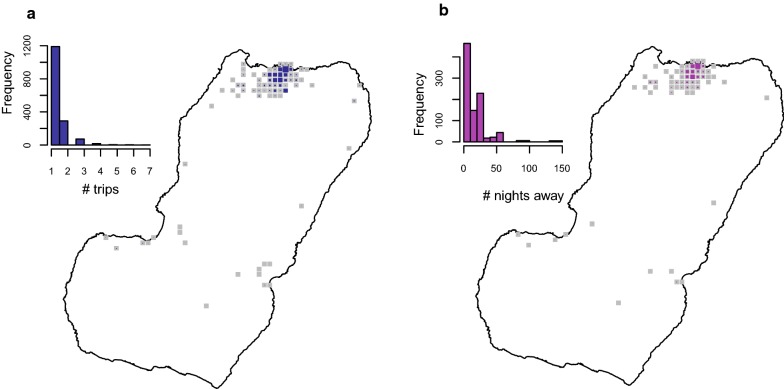
Frequency of travel and time at risk spent in mainland Equatorial Guinea. **a** Travel rate to mainland EG, or number of trips made within the 8 weeks preceding the MIS. The inset histogram illustrates the frequency distribution and the map shows the travel rate by area of residence expressed as the mean weighted by sample size. The grey boxes correspond to $1 \times 1$ km areas with data available and the blue boxes are proportional in size to the weighted mean of the travel rate. **b** Cumulative time spent in mainland EG by area of residence. The grey boxes are $1 \times 1$ km areas for which this information is available and the size of the purple squares is proportional to the maximum median man-nights spent abroad (60 nights). The distribution of nights away from home during trips to mainland is illustrated in the inset histogram

**Fig. 5:**
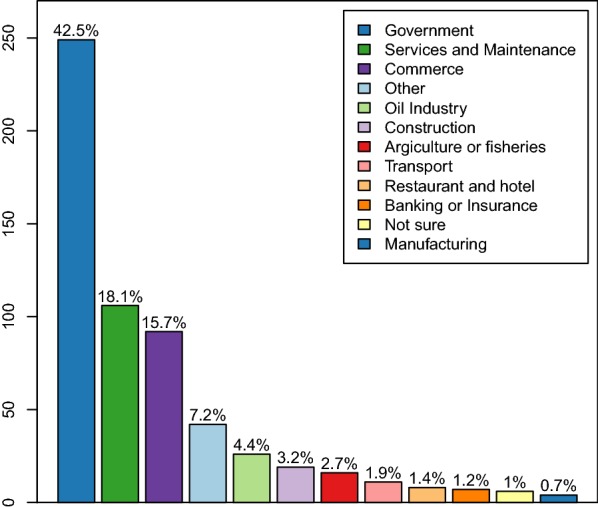
Frequency of travellers to mainland Equatorial Guinea, by job sector. Identifying high-risk travellers is critical for targeting specific groups

**Fig. 6:**
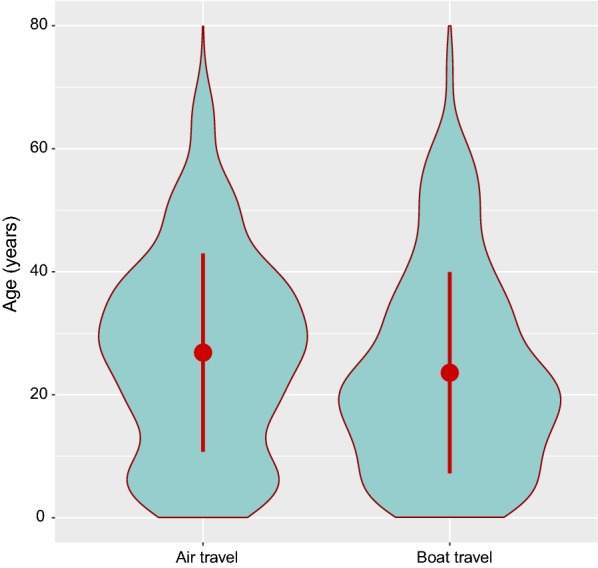
Age distribution of travellers to mainland Equatorial Guinea according to means of transportation. The violin plots illustrate the probability density functions and the red circle and bars the mean ± one standard deviation. The age structures of air and boat travellers are different, with a predominance of children and young adults travelling by boat and of older adults travelling by airplane

The modifications to the MIS questionnaire translated into substantial improvements to the travel data (Figs. 2, 3, 4, 5, 6). Overall, within the 8 weeks preceding the 2018 survey, 2394 trips were reported by 1423 travellers (10.3% of those surveyed) to 151 out of 306 different communities within Bioko and 1577 trips by 857 travellers (6.2% of individuals surveyed) to 15 districts in mainland EG. Figure 2 illustrates the improvement on the level of detail of on-island destinations compared to the spatial resolution available from previous MIS. The new data revealed that 74.1% of travellers to mainland EG were bound to only three districts (Bata, Ebibeyin and Mongomo) and 47.6% to a single district (Bata; Fig. 3). The data also showed that, on average, travellers to the mainland made 1.4 trips during the 8 week survey period, and remained 18 nights away per trip. Figure 4 shows that the cumulative time spent in mainland EG and the frequency of trips were considerably higher in Malabo residents. It was also possible to determine that 76.3% of travellers to mainland worked in only three job sectors (Fig. 5) and that 58.4% used air travel (Fig. 6).

By recording who is infected and who travels where with greater detail, the MIS travel data can be used to draw a synthetic picture of malaria transmission, which can account both for variability in FOI across different locations ($h_i$) as well as the time at risk spent while travelling to these locations ($p_{i,j}$). Local FOI can be calculated from MIS-derived estimates of *Pf*PR across Bioko Island and, for off-island locations, from *Pf*PR data from cross-sectional surveys [37] or *Pf*PR predicted with geostatistical models [17, 38].

Calculating a time at risk matrix requires data describing the frequency at which people travel from one location to another. The connectivity maps shown in Fig. 2 reflect the trips taken between locations and the improved spatial resolution of the 2018 MIS. The same can be done with mainland EG, where destinations can be treated as a single patch or as 15 separate patches (Fig. 3). Additionally, a time at risk matrix requires data describing the duration of travel, which was also included as a question in the 2018 MIS. The new MIS data revealed strong spatial patterns in the frequency of travel and the time spent travelling (Fig. 4), suggesting that ignoring the heterogeneities in travel behaviour might inaccurately reflect the truth of malaria exposure and transmission patterns among travellers.

**Fig. 7:**
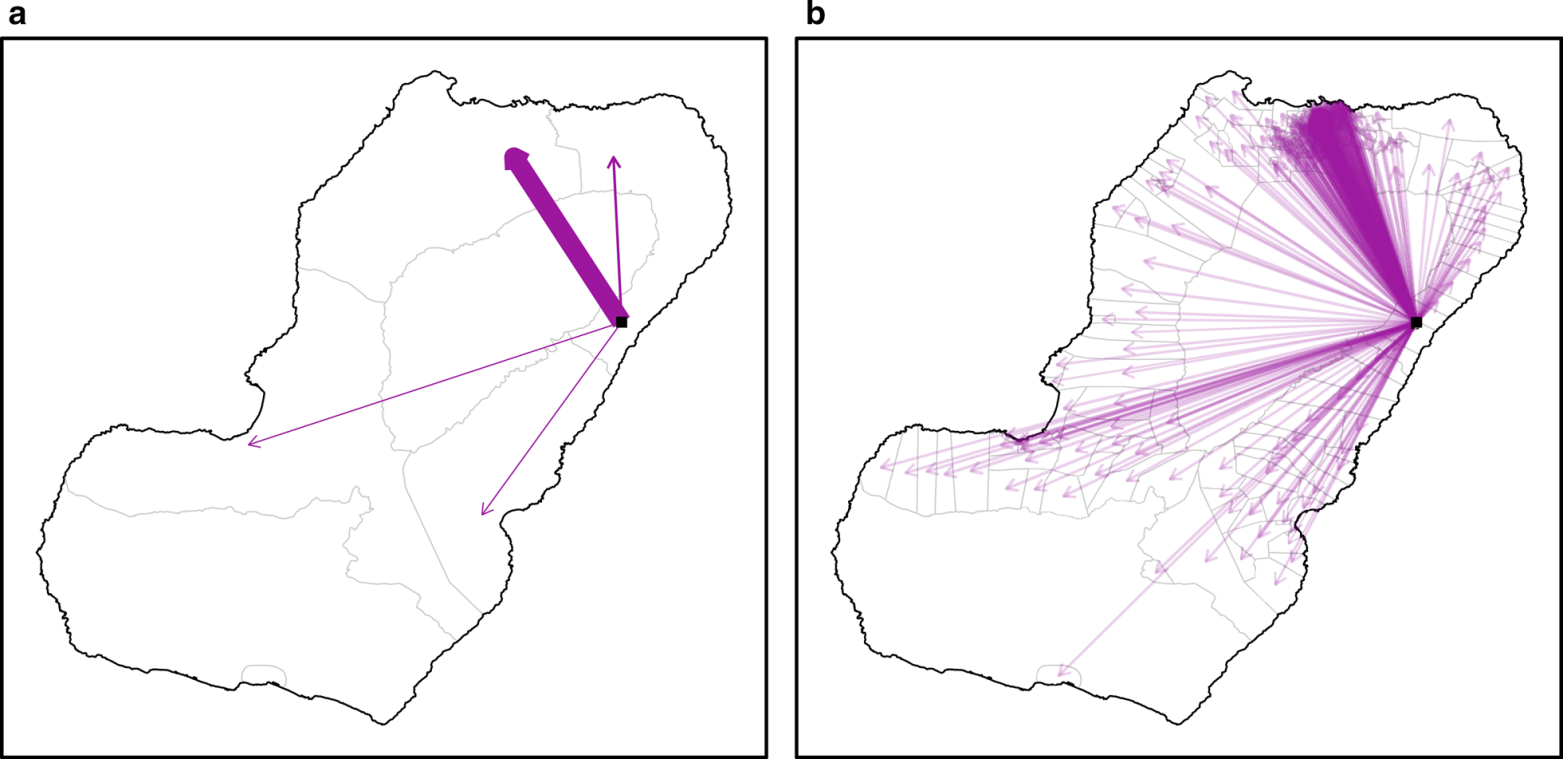
An example of a time at risk matrix for on-island human travel using data from MIS. The arrows illustrate the estimated flow of travellers from one area (black square) to all possible destinations documented at the second administrative level (2015–2017 MIS data, **a**) and at the fourth administrative level (2018 MIS data, **b**). The arrow thicknesses are proportional to the time at risk spent at each destination

An example of a time at risk matrix between locations within Bioko is shown in Fig. 7. For Fig. 7a, the times at risk were calculated using a gravity model [39, 40] fit to the number of trips to each second administrative unit reported in the MIS, combined with the assumption that the duration of on-island travel lasted 1 week, on average. For Fig. 7b, the times at risk were calculated using another gravity model fit to the number of trips to each fourth administrative unit, combined with the duration and frequency of travel reported in the 2018 MIS. This further illustrates the level of detail gained through the improved MIS travel data.

It is imperative to emphasise that without knowing both the trip frequency and the duration of travel it is very difficult to accurately characterise the amount of time at risk spent in different locations. As such, the new questions in the modified 2018 MIS make it possible to estimate each traveller’s overall FOI. All this information needs to be put together into a fine-grained mechanistic model of malaria transmission that includes three core elements: estimates of human malaria exposure within each geographically defined sub-population, estimates of parasite dispersion among sub-populations by mosquitoes, and estimates of parasite dispersion among sub-populations by humans. The spatial scale at which the sub-populations should be defined remains a critical unknown, but the patterns of mosquito dispersal from other studies provide an important guide [4, 5, 8]. All this is part of ongoing modelling work that will be used to guide the targeting of malaria interventions on Bioko Island.

## Conclusions

The success of malaria control and elimination requires an in-depth understanding of human movement and its role connecting demographic sources and sinks of malaria parasites [6]. If the flow of pathogens from sources to local sinks remains constant, reducing local $R_c$ below 1 may be insufficient to eliminate malaria. In the example of Bioko, persistent transmission hot spots make the island highly receptive to imported infections [30]. At the same time, malaria control in mainland EG is limited and parasite prevalence remains high. In a cross-sectional survey conducted in 2013 in Bata district, the most common destination for Bioko travellers, *Pf*PR in all ages fluctuated between 33.9% in urban and 58.9% in rural areas [37]. The strong connections of human movement between Bioko and the mainland determine a high vulnerability of Bioko to malaria importation. It is, therefore, essential to identify human mobility patterns that allow characterising these connections and quantifying the extent of malaria importation. An accurate and detailed picture of malaria connectivity would allow to design optimal combinations of control strategies to tackle both parasite importation and local transmission. In order to provide such a description of malaria connectivity, however, there is a need to know something about how and when people move between areas of differing endemicity. This is generally hindered by a paucity of human movement data. This paper argues that the integration of a travel component within MIS is straightforward and can turn these surveys into useful sources of human movement data.

There are some advantages of MIS over other sources of travel data. First, in many settings, MIS are routinely conducted and the resulting data can be readily available for processing and analyses. Second, MIS gather additional demographic information on individuals and households that can provide further insights, such as the motivations for travelling and demographic biases affecting travel (Figs. 5, 6) [41]. Third, crucially, MIS usually collect data on parasite prevalence in the population, providing an opportunity to investigate how the odds of travelling are linked to the odds of malaria infection. The experience on Bioko Island shows that simple modifications to the MIS questionnaire, tailored to fill specific information gaps, can boost the amount and quality of the information collected, critically improving the utility of MIS travel data. Table 1 summarises the potential application of the improved travel data on designing and planning adequate interventions against malaria importation on Bioko Island. As noted above, time at risk is determined by a combination of how frequently travellers go to and how long they spend at their destinations. If there are particular patterns in people who travel frequently and for extended periods of time, such as is observed on Bioko, then this information may be useful for recommending prophylaxis for departing travellers and increased surveillance of returning travellers. Moreover, if certain demographic groups are more likely to travel to high risk areas, then such information may also be useful for recommending drug prophylaxis or use of personal protection (e.g. LLINs) to those groups. Identifying the ports of entry and the demographics according to means of transportation is also useful for designing the most cost-beneficial border interdiction strategies. Finally, when feasible, malaria control at the destinations that represent the main sources of malaria importation would reduce the FOI *there* and hence reduce the risk of infection of travellers returning *here*. The collective contribution of controlling malaria importation through a combination of all or any of the above interventions is dependent on the effect size on the FOI. Current work is addressing this problem through developing human travel and transmission models calibrated largely through the data presented in this paper. The potential interventions listed are applicable to other areas, but they would need adapting to the specific contextual realities.

**Table 1:** Potential application of detailed travel data for planning interventions

Travel data improvement	Intervention strategy
Frequency of travel	Target specific interventions to frequent travellers, such as prophylaxis and protective measures
Time spent during trips	Identify areas where cumulative time at risk is higher and hence greater surveillance for ongoing transmission is granted
Greater geographic detail of destinations	Characterise the main sources of incoming parasites to guide adequate measures to control such foci
Characterising travellers by occupation/reasons for travel	Identify high risk groups to target at borders with specific interventions (e.g. travellers of certain professional occupation are prescribed prophylaxis during travel)
Identifying means of transportation	Guide optimal suite of interventions at borders; this can be enhanced by knowing the age structure of travellers (Fig. 6)

The MIS travel data suffer from some potential limitations. First, there is the problem of recall bias whereby individuals’ responses may not be an accurate reflection of their travel history. These biases are more likely to occur when wider time windows are used in the travel questions [9, 29, 30, 41]. For instance, if people are asked about their travel in the previous 2 weeks their responses will likely be less biased than when asked about trips taken in the past 8 weeks or 6 months. Second, responses provide a snapshot of individual travel making it difficult to estimate the seasonality and frequency of travel, therefore obscuring the time component of malaria connectivity. In other words, the risk of malaria infection at a given destination is also determined by the seasonality of transmission at that place. Importantly, one of the challenges of modelling malaria importation using human travel data is that malaria infections may have occurred before the time window during which travel was reported, and certain assumptions need to be made to account for this unknown [30]. The time window used in the history of travel questions is designed to widen this snapshot. Therefore, while a narrow time window can compensate for recall bias, it will compromise the amount of data that can be collected and the ability of the data to reflect the seasonal component and the frequency of travel. There is a need to find a balance when defining the time window that describes recent travel in order to optimise the amount of data available whilst minimising errors introduced by recall bias.

Other sources of travel data, namely call detail records (CDR) and global positioning systems (GPS) data loggers, have found application in the study of human mobility and infectious disease dynamics, including malaria [3, 7, 12, 13, 15, 42–51]. These data sets, however, tend to be considerably large and the data can prove overwhelmingly difficult to process and analyse as well as logistically hard to procure [9]. Moreover, these technologies suffer from their own inherent limitations and are not exempt of biases and technical difficulties [9, 43, 44, 50, 52]; therefore, they can be of limited use in certain settings. Wesolowski et al. compared travel data obtained from community surveys with CDR data in an area of Kenya where both data sets were available over the same time period [41]. They found that the volume of travel reported through the surveys was significantly lower than that evidenced through the CDR, which also provided higher spatio-temporal precision of travel patterns. The surveys, conversely, revealed demographic information about travellers that could not be captured by the CDR data. In certain circumstances, a combination of MIS and CDR/GPS data loggers can prove a good recipe for data collection [15].

Detailed travel questions in MIS can help narrow the information gap between MIS travel data and these more sophisticated technologies. Disaggregated information on travel destinations, time at risk spent away from home and frequency of travel is critical for assessing risk of infection whilst travelling and for describing malaria connectivity [6]. Consider the difference between the risk of parasite infection in a sporadic traveller spending one night in an area where malaria prevalence in the local population is 10% compared to that in a frequent traveller taking several trips of 2 weeks to an area where prevalence is 50%. The MIS data show that the latter is the case of many Bioko travellers visiting mainland EG, where they spend an average of around 3 weeks and where transmission intensity is high. Not having access to complete duration and frequency of travel data makes it difficult to characterise time at risk. At the same time, the added benefit for the spatial characterisation of malaria connectivity of having more precise information on the travel destination is intuitive. Such level of detail is normally offered by CDR and GPS data loggers and most certainly absent from MIS.

The example of the Bioko Island MIS proves unprecedented in that the questionnaire was adapted to specific information needs, which produced highly detailed travel data that are, in some ways, comparable in detail to and in other ways more comprehensive than the information potentially attainable through the more sophisticated approaches discussed above. These data were drawn from a large population sample on which parasitaemia was also measured, rendering them ideal for describing malaria connectivity. Alternative methods to investigate this have used travel history of malaria positive cases rather than of individuals surveyed in the community [53–55]. Such approaches are suitable for areas where local transmission has reached extremely low levels that make it feasible to identify most cases through active case detection. The use of MIS travel and prevalence data are better indicated for scenarios like Bioko Island, where there is significant heterogeneity of local residual transmission and where foci of relatively high prevalence remain active [30]. Such scenarios are commonplace in other countries and regions that have successfully reduced the local burden of malaria and start facing the challenge of controlling new infections acquired in higher transmission neighbouring or distant areas. The addition of molecular epidemiological techniques designed to assess the extent of parasite genetic mixing [14, 15] could prove the optimal prescription in these scenarios. Future MIS on Bioko Island aim to collect filter paper blood samples towards this purpose.

Malaria control on Bioko Island is unusual because it has benefited from sustained funding through a strong public-private partnership, a condition that is not ubiquitous across malaria endemic areas. As a result, substantial resources have been invested such as, for example, in developing and maintaining a comprehensive system of household enumeration that supports all interventions, including MIS, which translates into highly geographically resolved data [56]. Thanks to this funding, it has also been possible to sustain annual MIS that are used as a monitoring tool able to produce periodically updated, high quality information. Such certainly is not the reality of many countries facing scarcer resources for malaria control. It is worth emphasising, however, that MIS are used very widely and adapting their questionnaires to accommodate detailed travel data to inform interventions seems feasible, beneficial and cost effective. Therefore, the utility of MIS to convey travel, malariometric and demographic data with the aim to characterise malaria connectivity deserves more attention by malaria control and elimination programmes.

## Data Availability

The datasets used and analysed during the current study are available from the corresponding author on reasonable request.

## Abbreviations

MIS: malaria indicator survey
EG: Equatorial Guinea
*Pf*PR: *Plasmodium falciparum* parasite rate
FOI: force of infection
CDR: call detail records
GPS: global positioning systems
